# Salivary microbiota and IgA responses are different in pre-diabetic individuals compared to normoglycemic controls

**DOI:** 10.3389/fcimb.2025.1591285

**Published:** 2025-06-04

**Authors:** Nahoko Kato-Kogoe, Kouji Tsuda, Asako Kudo, Shoichi Sakaguchi, Michi Omori, Eri Komori, Mayu Ohmichi, Wataru Hamada, Shota Nakamura, Takashi Nakano, Junko Tamaki, Takaaki Ueno

**Affiliations:** ^1^ Department of Dentistry and Oral Surgery, Faculty of Medicine, Osaka Medical and Pharmaceutical University, Takatsuki, Japan; ^2^ Department of Hygiene and Public Health, Faculty of Medicine, Osaka Medical and Pharmaceutical University, Takatsuki, Japan; ^3^ Department of Microbiology and Infection Control, Faculty of Medicine, Osaka Medical and Pharmaceutical University, Takatsuki, Japan; ^4^ Department of Infection Metagenomics, Genome Information Research Center, Research Institute for Microbial Diseases, Osaka University, Suita, Japan

**Keywords:** pre-diabetes, microbiota, saliva, immunoglobulin A, 16S rRNA, IgA-seq

## Abstract

**Introduction:**

In recent years, changes in the oral microbiota of patients with type 2 diabetes mellitus (T2DM) have been increasingly recognized. The salivary microbiota may also be altered in pre-diabetes, which is the earliest stage of abnormal blood glucose regulation and a reversible stage preceding T2DM; however, its characteristics are poorly understood. Salivary immunoglobulin A (IgA) is a host defense factor central to the oral immune system and may play an important role in regulating the salivary microbiota. Given that alterations in immunoreactivity are observed in pre-diabetes, we hypothesized that the salivary IgA response may also be altered; however, limited knowledge exists regarding this. Therefore, in the present study, we aimed to evaluate the characteristics of salivary microbiota and IgA responses against salivary microbiota in individuals with pre-diabetes, comparing them to those in individuals with normoglycemia.

**Methods:**

Saliva samples were collected from 101 pre-diabetic individuals (PreDM group) and 101 age- and sex-matched normoglycemic controls (Normal group). Further, 16S rRNA metagenomic analysis was performed to compare bacterial microbiota composition. For each of the 19 saliva samples from the PreDM and Normal groups, IgA-enriched and IgA-nonenriched fractions were separated via magnetic-activated cell sorting, followed by 16S rRNA metagenomic analysis. The IgA index was calculated to evaluate the difference in the IgA response to each bacterium between the PreDM and Normal groups.

**Results:**

Bacterial species richness was significantly lower in the PreDM group than in the Normal group (observed operational taxonomic unit index, *p* = 0.042), and a difference between these groups was noted in the overall salivary microbiota structure (unweighted UniFrac distances, *p* = 0.009). Salivary IgA responses against several bacterial genera differed between the PreDM and Normal groups. Significantly higher IgA responses were noted against *Haemophilus* in the PreDM group, with lower responses against *Capnocytophaga*, *Corynebacterium*, and *Streptococcus* relative to those in the Normal group.

**Conclusions:**

Salivary microbiota and IgA responses differ between pre-diabetic individuals and normoglycemic controls. The current findings advance our understanding of the interaction between oral bacteria and host immune responses in patients with a poor glycemic status.

## Introduction

Type 2 diabetes mellitus (T2DM) is among the most prevalent metabolic diseases worldwide. It is characterized by insulin resistance and hyperglycemia, which can lead to systemic disorders and various complications. The earliest stage of abnormal blood glucose regulation is called pre-diabetes, a condition in which blood glucose levels are elevated but below the diabetic threshold (American Diabetes Association, 2022). Pre-diabetes is a significant risk factor for the development of T2DM as well as also other complications, such as cardiovascular and renal diseases (Mutie et al., 2020; Echouffo-Tcheugui and Selvin, 2021). The prevalence of pre-diabetes has increased significantly in recent decades, affecting approximately 7.5% of the world’s population, that is, approximately 374 million people (Rooney et al., 2023). As impaired insulin secretion and insulin resistance are associated with aging, the incidence and complications of T2DM are expected to increase as the population ages (Saeedi et al., 2019; Sinclair et al., 2020; Sun et al., 2022). Current evidence suggests that the prevention of T2DM and cardiovascular disease is most effective when conducted at the beginning of the disease process (Echouffo-Tcheugui and Selvin, 2021). Therefore, the timely detection of pre-diabetes is essential so that patients can begin to manage the disease as early as possible. Identifying the signatures of pre-diabetes will facilitate its comprehensive evaluation and effective management.

Diabetes and oral health have a clinically significant reciprocal relationship (Borgnakke, 2019). Patients with T2DM have an increased risk and susceptibility to oral infections, such as dental caries and periodontal disease, with local and systemic inflammation due to oral bacterial infection reducing glycemic control (Verhulst et al., 2019; Genco and Borgnakke, 2020). In recent years, changes in the oral microbiota of patients with T2DM have become evident (Hardinsyah et al., 2023). We have previously demonstrated that the salivary microbiota of patients with T2DM differs from that of healthy controls among older Japanese adults (Omori et al., 2022). Several reports have focused on salivary microbiota characteristics in pre-diabetic individuals (Saeb et al., 2019; Wang et al., 2019; Vieira Lima et al., 2022). However, these studies were relatively limited in sample size, and no consensus has been reached yet. Furthermore, as the salivary microbiota is known to vary by race, ethnicity, and age (Human Microbiome Project Consortium, 2012; Lira-Junior et al., 2018; Wu et al., 2020; Zaura et al., 2021), more age-matched studies are needed, which have yet been performed in previous research (Saeb et al., 2019; Wang et al., 2019; Vieira Lima et al., 2022).

Salivary immunoglobulin A (IgA) antibodies are host defense factors central to the oral immune system, acting at the gastrointestinal and respiratory tract frontlines (Marcotte and Lavoie, 1998). IgA plays a role in immune defense on mucosal surfaces, such as the intestines, lungs, non-urinary organs, and eyes, in addition to the oral cavity. Recently, it has become clear that IgA plays an important role not only in the elimination of pathogens, but also in the maintenance of commensal microbiota homeostasis (Bunker et al., 2017; Takeuchi and Ohno, 2022). IgA responses in the gut have been linked to specific diseases and conditions, such as inflammatory bowel disease (Palm et al., 2014), severe malnutrition (Kau et al., 2015), and aging (Sugahara et al., 2017). However, little is known regarding the response of salivary IgA to oral bacteria. Given that chronic inflammation and altered immune responses are considered to be associated with the pathogenesis of pre-diabetes (Zhou et al., 2018), we speculated that salivary IgA responses to the oral microbiota may be altered. Therefore, in the present study, we investigated the characteristics of the salivary IgA response to oral microbiota in pre-diabetic individuals using a combination of magnetic-activated cell sorting (MACS) and 16S rRNA gene sequencing (IgA-SEQ). Characterizing the salivary IgA response in pre-diabetic individuals is important for our understanding of the oral environment, not only in terms of microbiota, but also in terms of biological responses.

In the present study, we evaluated the characteristics of salivary microbiota and IgA responses against salivary microbiota in pre-diabetic individuals, comparing them to those in normoglycemic controls.

## Materials and methods

### Participants

The study was conducted in accordance with the guidelines of the Declaration of Helsinki and its latest revision. This study was approved by the Ethics Committee of Osaka Medical and Pharmaceutical University, Takatsuki, Japan (Approval No. 2145). Written informed consent was obtained from all participants.

The study population included 101 pre-diabetic individuals (33% male, 65–87 years old) and 101 normoglycemic controls matched by age, sex, and hypertension status. Pre-diabetic individuals were defined as having fasting plasma glucose (FPG) levels of 100–125 mg/dL or HbA1c (NGSP) 5.6–6.4% (PreDM group), and normoglycemic controls were defined as having FPG <100 mg/dL and HbA1c (NGSP) <5.6% (Normal group). Hypertension was defined as a systolic blood pressure ≥ 140 mmHg, or a diastolic blood pressure ≥ 90 mmHg, or the use of antihypertensive drugs. The study participants were 443 residents who participated in the Takatsuki study conducted in the period between May 2018 and November 2019. The Takatsuki Study was a prospective cohort study of older residents aged ≥65 years in Takatsuki City that aimed to elucidate the relationship between oral health and various systemic diseases. Patients with diabetes mellitus, defined as those with HbA1c > 6.5%, those using oral antidiabetic drugs or on insulin therapy, or participants with a body mass index (BMI) ≥ 30 BMI kg/m^2^ were excluded. In addition, subjects receiving treatment for malignancy, rheumatoid arthritis, severe renal or hepatic disease, stroke, and ischemic heart disease, and those taking antimicrobials at least one month prior to sample collection were excluded based on self-report and medical information.

### Saliva sample collection and oral examination

Saliva samples were collected, and oral examinations were performed according to our previously reported methods (Omori et al., 2021). Briefly, saliva samples were collected from participants in the morning, at least 2 h after brushing or eating, using the SalivaBio^®^ oral swab and swab storage tube (Salimetrics, Irvine, PA, USA) saliva collection system prior to oral examination. Samples were frozen immediately after collection and stored at -80°C until DNA extraction. All participants underwent a full-mouth clinical examination by a dental specialist.

### Quantification of IgA in saliva samples

The amount of secretory IgA in saliva samples was quantified using a salivary secretory IgA indirect enzyme immunoassay kit (Salimetrics LLC, Carlsbad, CA, USA) according to the manufacturer’s instructions.

### DNA extraction, 16S rRNA sequencing, and taxonomic classification

DNA extraction, 16S rRNA sequencing, and taxonomic classification were performed as previously described (Kato-Kogoe et al., 2023). Briefly, samples were homogenized, DNA was extracted using GENE PREP STAR PI-480 (Kurashiki Spinning Co., Ltd., Osaka, Japan) according to the manufacturer’s instructions, and DNA concentration was measured. The V1-V2 region of the 16S rRNA gene was PCR-amplified, and the library was prepared by adding Index (Illumina, San Diego, CA, USA) and checked for quality. A 250-bp paired-end sequence was generated in 500 cycles using MiSeq Reagent Kit v2 (Illumina). An average of 28,758 sequence reads were denoised using DADA2 in Quantitative Insights into Microbial Ecology 2 (QIIME2) version 2020.08 and quality filtered. The minimum depth cutoff for rarefaction was set at 10,000, and each OUT was assigned to the curated Greengenes 13_8 reference database.

### Evaluation of IgA response to salivary microbiota

To characterize the IgA response to salivary microbiota, antibody-based bacterial sorting combined with 16S ribosomal RNA gene sequencing (IgA-SEQ), a modified version of a previously described method (Palm et al., 2014), was performed. Among the participants described above, pre-diabetic subjects (n = 19) and age-matched normoglycemic controls (n = 19) were selected. IgA-enriched and IgA-nonenriched fractions were separated from their saliva. The baseline characteristics and laboratory data of the participants are presented in **Supplementary Table S1**.

Saliva samples were stained with phycoerythrin (PE)-labeled mouse anti-human IgA (Miltenyi Biotec Cat# 130-093-128), whereafter anti-PE magnetically-activated cell sorting beads (Miltenyi Biotec Cat# 130-105-639) and MS columns (Miltenyi Biotec Cat# 130-042-201) were used to separate and collect IgA-enriched (IgA(+)) and IgA-nonenriched (IgA(-)) fractions. For each of the presort, IgA(+), and IgA(-) fractions, DNA extraction, 16S rRNA sequencing, and taxonomic classification were performed as described in the previous section.

To compare the levels of IgA responses for specific bacteria in the PreDM and Normal groups, the IgA index was calculated for the bacterial genera present in more than 50% of the participants in each fraction. The IgA index was calculated as the log ratio of the difference between IgA(+) and IgA(-) bacteria over the sum of IgA(+) and IgA(-) bacteria, (IgA index = − (log(IgA(+) taxon) − log(IgA(-) taxon))/(log(IgA(+) taxon) + log(IgA(-) taxon), according to previous reports (Kau et al., 2015; Planer et al., 2016).

### Statistical analysis

Between-group comparisons of patient characteristics were performed using the Wilcoxon rank-sum test and Fisher’s exact test, as appropriate. The statistical software R programming version 4.0.0 was used for database construction and data analysis. Statistical significance was set at *p* < 0.05.

To determine the richness and evenness of bacterial communities, alpha-diversity was assessed using the observed operational taxonomic unit (OUT) index and Shannon index and was compared among groups using the Kruskal–Wallis test. Beta-diversity among groups was assessed using phylogenetic tree-based indices, unweighted and weighted UniFrac distance metrics, and visualized using Principal Coordinate Analysis (PCoA). In addition, the significance of compositional differences between groups was assessed using permutational multivariate analysis of variance (PERMANOVA). The QIIME2 software was used for these analyses. To detect bacteria with different abundance ratios among groups, the linear discriminant analysis effect size (LEfSe) algorithm was used. The alpha parameter for LEfSe’s pairwise test was set to 0.05, and the threshold for the log score was set to 2.0.

## Results

### Characteristics of the participants

There were no significant differences in the basic characteristics, oral status, current comorbidities, or laboratory values between participants in the PreDM (n = 101) and Normal groups (n = 101), except for parameters related to glucose metabolic status and BMI (**Table 1**). Salivary IgA concentration averaged 353.9 ± 265.2 μg/mL in the PreDM group and 334.0 ± 285.6 μg/mL in the Normal group, with no significant difference between the two groups (**Supplementary Figure 1**).

**Table 1:** Baseline characteristics and laboratory data of the study population (n = 202).

Variables	PreDM (n = 101)	Normal (n = 101)	*p*-value
Characteristics			
Age (years)	74 (71–78)	75 (71–79)	0.806
Sex (male, n (%))	33 (33%)	33 (33%)	> 0.999
BMI (kg/m^2^)	22.5 (20.2–24.6)	21.8 (19.7–23.2)	0.046
Smoking status (n (%))			
Never smoker	77 (76%)	71 (70%)	0.423
Ex-smoker	21 (21%)	23 (23%)	
Current smoker	3 (3.0%)	7 (6.9%)	
Oral condition			
Number of teeth (n (%))			
< 10	6 (5.9%)	12 (12%)	0.372
10-19	18 (18%)	17 (17%)	
≥ 20	77 (76%)	72 (71%)	
Denture wearing (n (%))	42 (42%)	41 (41%)	> 0.999
Salivary flow rate (g/min)	1.3 (0.9–1.9)	1.2 (0.8–2.1)	0.712
Laboratory data			
HbA1c (NGSP, %)	5.8 (5.6–5.9)	5.3 (5.2–5.4)	< 0.001
FPG (mg/dL)	97 (90–103)	90 (85–94)	< 0.001
CRP (mg/dL)	0.03 (0.01-0.06)	0.02 (0.01-0.04)	0.167
Current comorbidities			
Hypertension ^a^ (n (%))	59 (58%)	59 (58%)	> 0.999
Dyslipidemia ^b^ (n (%))	57 (56%)	49 (49%)	0.260
Osteoporosis (n (%))	15 (15%)	18 (18%)	0.568
Tumor (n (%))	0 (0%)	0 (0%)	–
Stroke (n (%))	0 (0%)	0 (0%)	–
Ischemic heart disease (n (%))	0 (0%)	0 (0%)	–
Pulmonary disease (n (%))	4 (4.0%)	3 (3.0%)	>0.999
Rheumatoid arthritis (n (%))	0 (0%)	0 (0%)	–
Insomnia (n (%))	8 (7.9%)	6 (5.9%)	0.580
Depression (n (%))	1 (1.0%)	1 (1.0%)	>0.999

PreDM, prediabetes; BMI, body mass index; FPG, fasting plasma glucose; CRP, C-reactive protein.

Results are expressed as the median (1st–3rd quartiles) or percentage. *p*-values are obtained using Wilcoxon rank sum test or Fisher’s exact test.

a Hypertension is defined as blood pressure ≥ 140/90 mmHg or use of anti-hypertensive drugs.

b Dyslipidemia is defined as low-density lipoprotein ≥ 140 mg/dL, high-density lipoprotein < 40 mg/dL, and triglycerides ≥ 150 mg/dL, or the use of anti-dyslipidemic drugs.

### Differences in salivary microbiota between PreDM and Normal groups

Salivary bacteria with relative abundances greater than 0.1% were classified into 11 phyla, 19 classes, 37 orders, 70 families, and 122 genera. At the genus level, 94 genera were present in the PreDM group, three of which were absent in the Normal group. In contrast, 119 genera were present in the Normal group, 27 of which were absent in the PreDM group. Forty genera were present in at least 50% of subjects in both groups, with 38 genera shared between the groups. The 20 most abundant genera in the PreDM and Normal groups accounted for 88.32% and 87.00% of the total genera abundance in the two groups, respectively (**Supplementary Table S2**).

**Figure 1:**
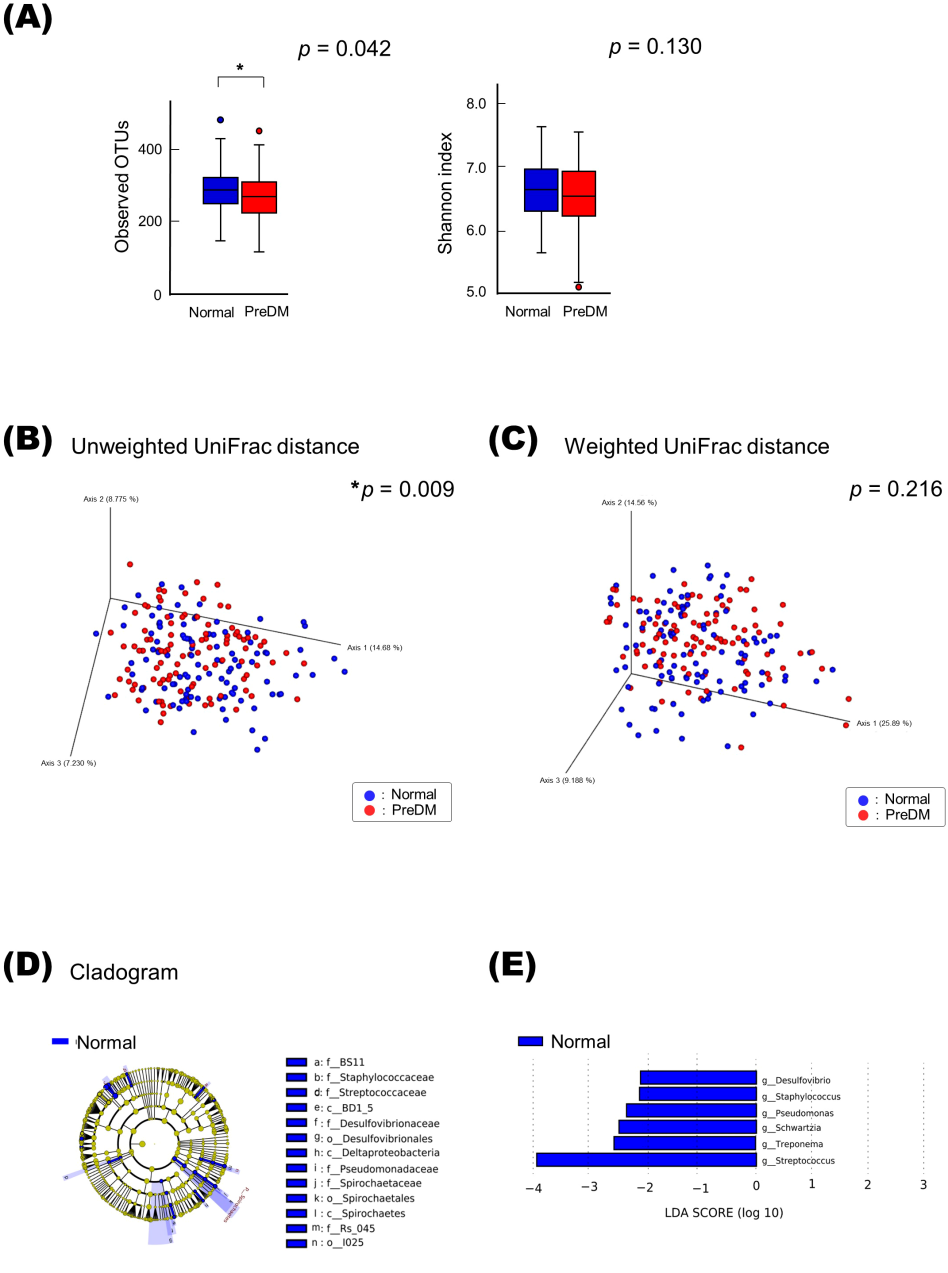
Differences in salivary microbiota between the PreDM and Normal groups. Alpha-diversity of the salivary microbiota **(A)**. Operational taxonomic unit (OTU) and Shannon indices in the Normal (n = 101, blue) and PreDM (n = 101, red) groups. *p < 0.05, compared among groups using the Kruskal–Wallis test. Beta-diversity of salivary microbiota. Unweighted **(B)** and weighted UniFrac distances **(C)**. Principal coordinate analysis (PCoA) plots for samples from 101 participants in the Normal group (blue) and 101 participants in the PreDM group (red). *p < 0.05, compared between groups using PERMANOVA with 999 permutations. Differentially abundant bacterial genera between the Normal and PreDM groups were identified using linear discriminant analysis effect size (LEfSe). Cladograms of differentially abundant bacterial taxa, with each layer representing a different taxon **(D)**. The enriched taxa in the Normal group (blue) are presented in the cladogram, while such were not found in the PreDM group. The central point represents the root of the tree (bacteria), and each ring represents the next lower taxonomic level (phylum to genus: p, phylum; c, class; o, order; f, family; g, genus). Histogram of the linear discriminant analysis (LDA) scores for differentially abundant bacterial taxa between Normal and PreDM groups **(E)**. LDA scores ≥ 2.0 are shown. Blue represents significantly abundant taxa in the Normal group compared to the PreDM group.

Analysis of alpha diversity in the salivary microbiota among PreDM and Normal groups revealed that bacterial species richness was significantly reduced in the PreDM group compared to that in the Normal group (observed OTU index, *p* = 0.042, **Figure 1A**), and there was no significant difference in bacterial evenness (Shannon index, *p* = 0.130). The PCoA plots, based on the unweighted UniFrac distance metric, revealed differences in the overall structure of the salivary microbiota between the Normal and PreDM groups in a three-dimensional space (**Figure 1B**). The difference in microbiota structure between the two groups was validated using PERMANOVA based on unweighted UniFrac data (999 permutations, *p* = 0.009). No statistically significant differences were observed in microbial structure between the two groups using the weighted UniFrac distance metric (*p* = 0.216; **Figure 1C**).

Bacterial genera that differed in abundance between the PreDM and Normal groups were identified using LEfSe analysis. The cladogram in **Figure 1D** shows the taxa that differed significantly between the two groups in the taxonomic hierarchy from phylum to genus. At the genus level, the Normal group showed a significantly increased abundance of *Streptococcus* compared to that in the PreDM group (**Figure 1E**).

### Differences in microbiota between IgA-enriched and IgA-nonenriched fractions

**Figure 2:**
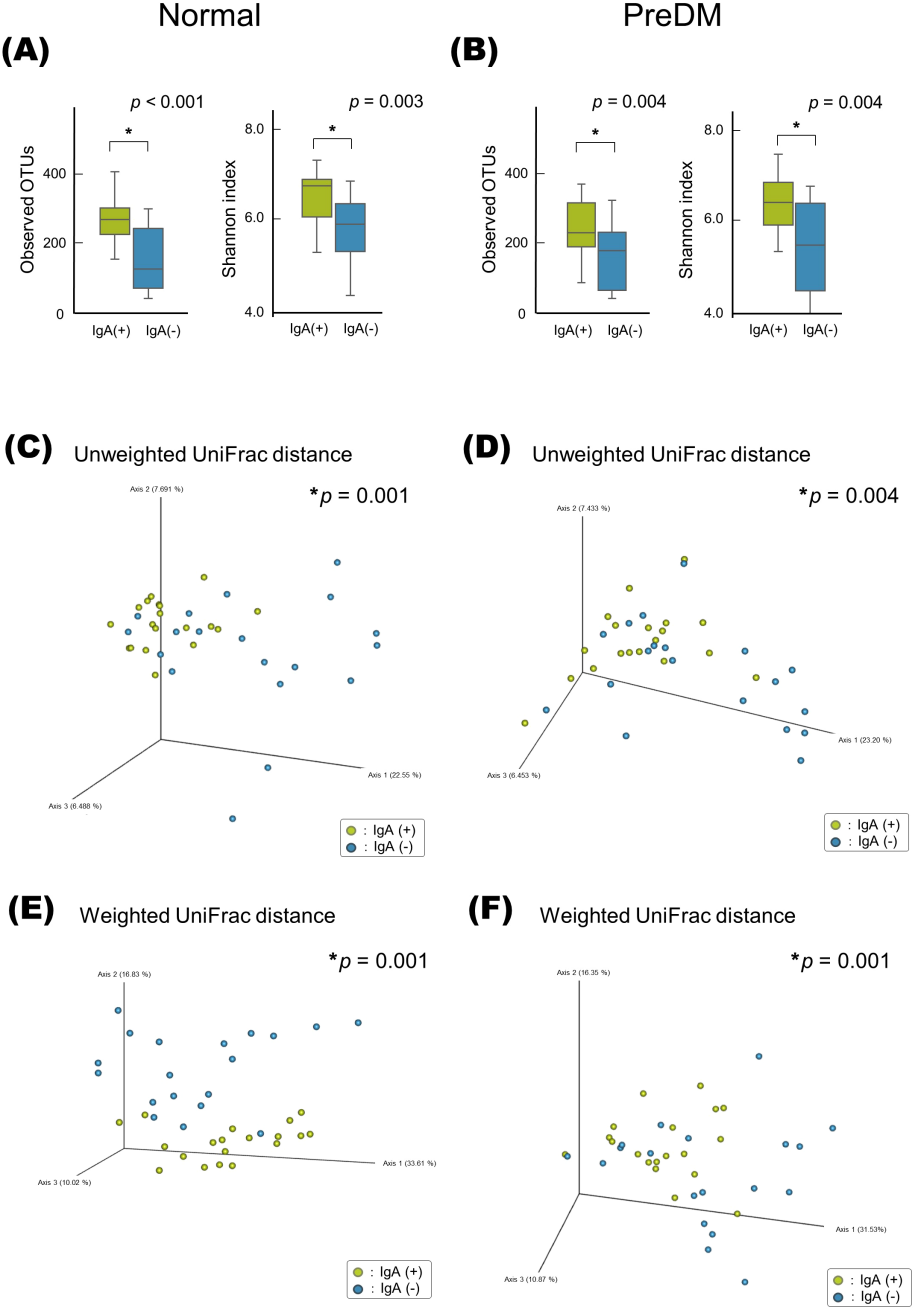
Alpha- and beta-diversity of microbiota in IgA-enriched and IgA-nonenriched fractions. Alpha-diversity of salivary microbiota in the Normal **(A)** and PreDM **(B)** groups. Operational taxonomic unit (OTU) and Shannon indices in the IgA-enriched (IgA (+)) and IgA-nonenriched (IgA (-)) fractions. *p < 0.05, compared among groups using the Kruskal–Wallis test. Beta-diversity of salivary microbiota. Unweighted UniFrac distances of Normal **(C)** and PreDM **(D)**, and Weighted UniFrac distances of Normal **(E)** and PreDM **(F)**. Principal coordinate analysis (PCoA) plot for 19 samples of IgA (+) and (IgA **(-)** fractions. *p < 0.05, compared between groups using PERMANOVA, 999 permutations.

The alpha-diversity of the IgA-enriched fraction was significantly higher than that of the IgA-nonenriched fraction in both the Normal and PreDM groups, respectively (**Figures 2A, B**). PCoA with unweighted and weighted UniFrac distances demonstrated that the microbiota differed between the IgA-enriched and IgA-nonenriched fractions. This difference in composition between the two groups was validated via PERMANOVA based on unweighted and weighted UniFrac distance metrics (*p* < 0.01; **Figures 2C-F**).

**Figure 3:**
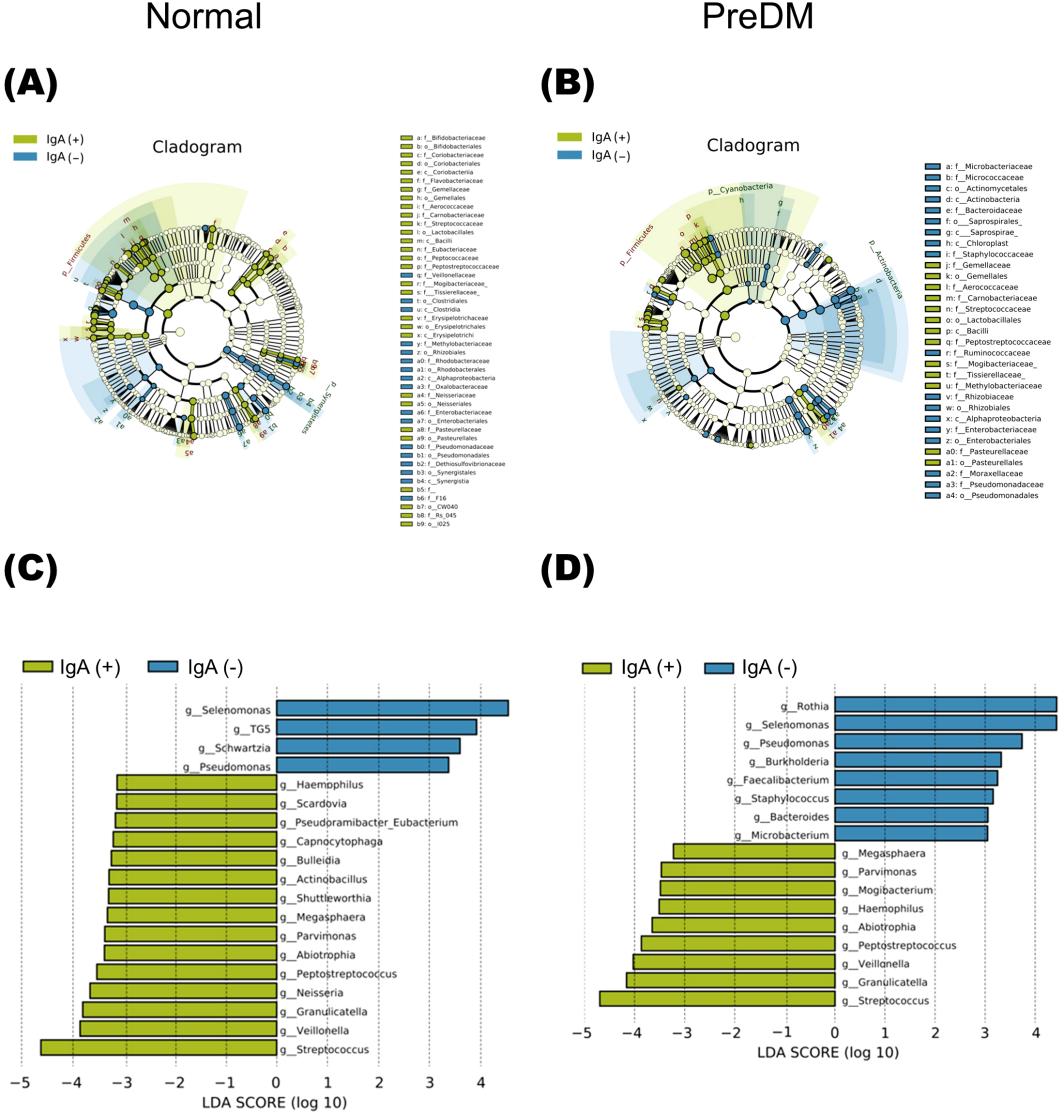
The differentially abundant bacterial genera between IgA-enriched and IgA-nonenriched fractions identified by linear discriminant analysis effect size (LEfSe). Cladogram of differentially abundant bacterial taxa, where each layer represents a different taxon in the Normal **(A)** and PreDM **(B)** groups. The enriched taxa in the IgA-enriched (IgA (+)) and IgA-nonenriched (IgA (-)) fractions are presented in the cladogram. Histogram of the linear discriminant analysis (LDA) scores for differentially abundant bacterial taxa between IgA (+) and IgA **(-)** fractions from Normal **(C)** and PreDM **(D)** groups. LDA scores ≥ 3.0 are shown. Yellow represents significantly abundant taxa in the IgA (+) fraction compared to those in the IgA **(-)** fraction. Blue represents significantly abundant taxa in the IgA **(-)** fraction compared with those in the IgA (+) fraction.

The LEfSe analysis presented in **Figure 3** revealed that *Rothia* was abundant in the IgA-nonenriched fraction in the PreDM group. In the Normal group, *Neisseria* and *Capnocytophaga* were abundant in the IgA-enriched fraction, and *Schwartzia* was more abundant in the IgA-nonenriched fraction. In both the Normal and PreDM groups, *Streptococcus*, *Veillonella*, *Granulicatella*, *Peptostreptococcus*, and *Haemophilus* were abundant in the IgA-enriched fractions, whereas *Selenomonas* and *Pseudomonas* were abundant in the IgA-nonenriched fractions. These results indicate that IgA responses vary among bacterial genera and are influenced by glucose metabolic status.

### Differences in IgA responses to specific bacteria between the PreDM and normal groups

**Figure 4:**
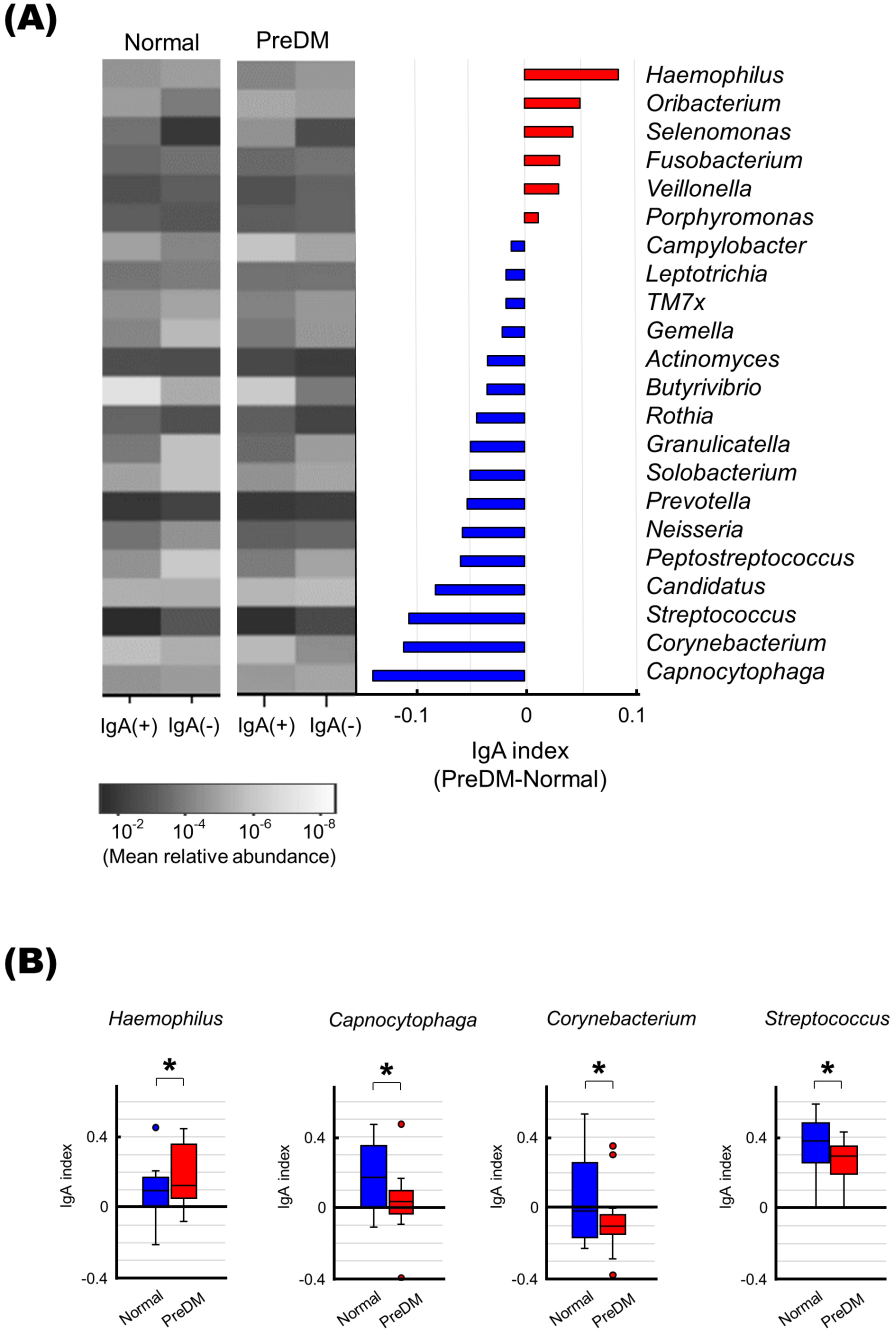
Differences in IgA responses to specific bacteria in the PreDM and Normal groups. **(A)** The left panel shows a heat map showing the mean relative abundance of 22 bacterial genera present in more than 50% of the participants in each fraction. IgA-enriched [IgA (+)) and IgA-nonenriched (IgA (-)] fractions of saliva from the Normal and PreDM groups (n = 19 each) are shown. The bar graph shows the IgA index difference between PreDM and Normal groups. IgA indexes are shown in order of size in PreDM. Red indicates bacterial genera with a larger IgA index in PreDM, blue indicates bacterial genera with a larger IgA index in Normal. **(B)** Boxplots of IgA index for each bacterial genus for the normal and PreDM groups are shown. *p < 0.05, compared between groups.

To evaluate differences in IgA responses to each bacterium between the PreDM and Normal groups, the IgA index was calculated for the 22 bacterial genera present in more than 50% of the subjects for each fraction. Bacterial genera associated with the PreDM and Normal groups were identified (**Figure 4A**). *Haemophilus* had a significantly higher IgA index in the Pre-DM group than in the Normal group (*p*<0.05, **Figure 4B**). *Capnocytophaga*, *Corynebacterium*, and *Streptococcus* had lower IgA indices in the PreDM group than in the Normal group (*p*<0.05, **Figure 4B**). These results showed differences in IgA responses to several bacterial genera between the PreDM and Normal groups.

## Discussion

In this study, we showed that the salivary microbiota of pre-diabetic individuals differs from that of age- and sex-matched normoglycemic controls. We also demonstrated that salivary IgA responses differ among bacterial genera. Furthermore, we revealed that the characteristics of the bacteria-specific IgA responses to salivary microbiota differ between the pre-diabetic and the normoglycemic individuals. These data suggest that glycemic control status may be related to the salivary microbiota and specific IgA responses. This advances our understanding of the interaction between oral bacteria and host immune responses in patients with a poor glycemic status.

Analysis of the alpha-diversity of salivary microbiota showed that species richness was significantly lower in the PreDM group than in the Normal group. This decreasing trend in microbial diversity is consistent with the results reported in previous studies (Saeb et al., 2019; Yang et al., 2020). The decreased species richness may be a result of poor glycemic control, which is a pathological condition that increases the concentration of glucose in the saliva and alters the availability and concentration of nutrients required for specific bacterial growth (Goodson et al., 2017; Longo et al., 2018). In contrast, some reports on diabetic patients have shown no difference in diversity or even the opposite trend, where diversity is higher in patients with diabetes than in healthy controls. Our previous report showed no difference in diversity between diabetic and healthy elderly Japanese (Omori et al., 2022). One possible cause of such discrepancies may be the subjects’ periodontal status. Although oral microbial diversity is reduced under condition of poor glycemic control, it increases in patients with diabetes with advanced periodontal disease compared to that of individuals with healthy periodontal tissue (Sabharwal et al., 2019). Therefore, oral conditions, including the severity of periodontal disease, may influence the diversity of the oral microbiota in patients with diabetes. In our study, there were no differences in oral status between the two groups, which may explain the decreased microbial diversity observed in the PreDM group.

LEfSe analysis revealed that the abundance of *Streptococcus* was higher in the normoglycemic control group than in the pre-diabetic group, which is consistent with a previous study (Rungrueang et al., 2021), while the opposite trend has also been reported (Wang et al., 2019). By contrast, many studies have indicated that patients with diabetes have an increased abundance of *Streptococcus* (Matsha et al., 2020; Wei et al., 2020; Liu et al., 2021). This may be explained by a report showing that a longer duration of diabetes mellitus in elderly individuals is associated with a higher abundance of *Streptococcus* (Zeng et al., 2024). There are also reports that persistent high blood glucose levels increase the abundance of *Streptococcus* (Goodson et al., 2017). The abundance of *Streptococcus* in patients with diabetes may be a compensatory condition observed in prolonged high blood glucose levels. This is because Firmicutes, including *Streptococcus*, are involved in facilitating energy metabolism, improving insulin sensitivity, and exerting anti-inflammatory effects, through butyrate production (Rauf et al., 2022). Therefore, the observation that the abundance of *Streptococcus* is lower in the pre-diabetic phase—the earliest stage of abnormal glucose regulation—than in the normoglycemic group is notable.

In recent years, the interaction between IgA and bacteria in the intestinal tract, which may reflect important immune-bacterial interactions in the maintenance or disruption of gut microbiota homeostasis, has been increasingly investigated (Bunker and Bendelac, 2018; Weis and Round, 2021). IgA is a major immune factor in the mucosa and plays an important role in bacterial elimination and colonization in a context-dependent manner (Moor et al., 2017; Donaldson et al., 2018; Huus et al., 2021). The oral cavity is an entry site for bacteria and is critical in that it is the first point of contact with the oral mucosa and the starting point of the immune response. Therefore, research on the interaction between IgA and oral bacteria is also increasing (Carpenter, 2020). However, little is known about the response of IgA to oral microbiota. Thus, in this study, we evaluated the IgA response of salivary microbiota.

In the present study, the bacterial composition in the IgA-enriched fraction were significantly different from those in the IgA-nonenriched fraction, and the IgA response was specific to the bacterial genera present in the salivary microbiota. This finding is consistent with a previous report (Simón-Soro et al., 2015). Notably, *Streptococcus*, *Veillonella*, and *Haemophilus*, which induced strong IgA responses in this study, are bacterial genera crucial for the initial formation of dental plaque biofilms (Mark Welch et al., 2016; Borisy and Valm, 2021). The strong IgA responses against *Haemophilus* and *Peptostreptococcus* as well as the weak IgA responses against *Selenomonas*, which were noted in both the Normal and PreDM groups in our study, were in line with recently reported salivary IgA biomes (Brown et al., 2020). However, as the number of participants is small—24 in the study by Brown et al. (2020) and 38 in our study—it is necessary to confirm this in a larger cohort. Nevertheless, the specificity of the IgA response for these bacteria may be related to the maintenance of oral microbiota by limiting or promoting bacterial colonization (Carpenter, 2020).

The results of our study revealed a specific bacterial population, against which the salivary IgA response differs between individuals with prediabetes and those with normoglycemia. These bacteria, such as *Haemophilus*, *Capnocytophaga* and *Corynebacterium*, could not be identified by comparing their relative abundance alone. Although the factors that contribute to the interaction between IgA and microbiota are poorly understood, there is increasing knowledge about how this interaction varies in across specific diseases, environmental factors, and nutritional conditions (Huus et al., 2021; Takeuchi and Ohno, 2022). In patients with inflammatory bowel disease, spondyloarthritis, and multiple sclerosis, IgA responses to specific intestinal bacteria are associated with disease severity (Palm et al., 2014; Gill et al., 2022; Gupta et al., 2025). The IgA response to the intestinal microbiota is also altered by aging, bariatric surgery, and the administration of antimicrobial agents (Sugahara et al., 2017; Scheithauer et al., 2021, 2022). In addition, nutritional status alters the IgA response to the gut microbiota, and changes in the IgA response to diet have been reported in mice (Kau et al., 2015; Huus et al., 2020; Tsuruta et al., 2023). Although IgA responses in metabolic disease are associated with dysbiosis and dysfunction of the intestinal microbiota (Klag and Round, 2021), little is known about the oral microbiota. Our findings on the interaction between IgA and the oral microbiota, which is altered in association with abnormal blood glucose levels, suggest that further investigation may identify microbial components that interact with the host immune system in the context of T2DM.

The relative abundance of *Haemophilus* did not differ between the PerDM and Normal groups, whereas the IgA response was significantly more potent in the PreDM group than in the Normal group in our study. A strong salivary IgA response to *Haemophilus* being associated with poor glycemic control is notable. Because the abundance of intestinal *Haemophilus* is increased in patients with IgA deficiency (Fadlallah et al., 2018; Sterlin et al., 2019), suggesting the importance of IgA in regulating the colonization of *Haemophilus* in the gut. Therefore, a strong salivary IgA response may eliminate this bacterium and affect the oral microbiota associated with poor glycemic control and T2DM. In fact, in our previous study, the abundance of *Haemophilus* in the salivary microbiota was lower in the T2DM group than in the normal group (Omori et al., 2022). In contrast, the PreDM group exhibited weaker IgA responses to *Capnocytophaga* and *Corynebacterium* than those of the Normal group in the present study. In previous studies, *Capnocytophaga* and *Corynebacterium* were reported to increase in hyperglycemic subjects (Ganesan et al., 2017; Graves et al., 2019). Thus, it is possible that decreased IgA responses is associated with an increase in these bacteria among salivary microbiota in PreDM in the current study. Furthermore, the relative abundance of *Streptococcus* was lower in the PerDM group than in the Normal group, and the IgA response was also weaker in the PreDM group than in the Normal group. IgA recognizing specific epitopes of *S. mitis*, *S. oralis*, and *S. mutans* has been reported to mediate bacterial colonization of oral mucosal surfaces (Carpenter, 2020). Thus, a decreased IgA response to *Streptococcus* may be associated with a reduced abundance of these genera in the PreDM group in the present study. Collectively, our results suggest that the salivary IgA response may be related to the composition of the salivary microbiota, affecting the proportion of specific bacteria present. The role of IgA in the regulation of the microbiota warrants further investigation.

Our study has some limitations. First, all study participants were elderly Japanese people living in Takatsuki City. Therefore, our results may not be generalizable to other populations. The background of the participants, such as age, ethnicity, and food culture, are factors known to influence salivary microbiota and IgA responses and may disrupt the study results if not addressed appropriately. In addition, the criteria used for selecting participants with prediabetes are also an important factor in interpreting the present results. In this study, PreDM was defined based on FPG and HbA1c (NGSP) laboratory values, based on Japanese guidelines, as the participants in this study were of Japanese ethnicity. However, diagnostic guidelines for prediabetes vary widely worldwide (Pragati and Paolo, 2025), with the glucose tolerance test recommended for the detection of IGT and diagnosis of prediabetes. Therefore, future studies should also carefully consider this factor. Furthermore, the present study did not account for the influence of confounding factors such as oral hygiene and periodontal status, which may affect the diversity and microbiota composition, owing to the lack of sufficient information on these factors. Given the correlation between diabetes and periodontal disease, these factors should be considered in future studies. Moreover, the influence of diet is also an important confounding factor in this study. Diet plays an important role in the development of hyperglycemia and prediabetes, with its influence on the microbiota becoming increasingly evident in recent years. Specifically, dietary interventions affect the oral and gut microbiota in individuals with prediabetes (Shoer et al., 2023). Moreover, diet-induced bacterial adaptation via IgA recognition in the gut has been reported in animal studies (Huus et al., 2020). Therefore, the effect of diet on the microbiota and IgA responses of PreDM participants should be considered in future studies. Overall, the method used in this study does not allow for an analysis that considers all confounding factors. Nonetheless, the results of this preliminary study warrant validation in a large-scale study in the future. Second, we evaluated the microbiota using 16S rRNA metagenomic analysis. This method emphasizes species composition and community diversity, assessing the relative abundance of bacteria rather than their absolute abundance. Therefore, it is necessary to measure the total abundance of these bacteria to further characterize the IgA response. Third, although salivary IgA secretion is altered by a variety of factors, including stress, aging, and circadian rhythms (Engeland et al., 2016; Castro-Quintas et al., 2023), little is known about the factors affecting the function of IgA on bacteria. In this study, participants were matched for age, and saliva collection was limited to the morning to ensure that there were no differences in salivary IgA secretion between groups. However, more extensive studies are needed to better understand how the effect of salivary IgA on the microbiota is related to glycemic status. Taken together, this preliminary study should be further validated to confirm our observations and the relationship between the salivary IgA response, glycemic status, and oral microbiota. The changes in oral bacterial composition identified in this study may be associated with diseases related to oral bacteria, such as dental disease and the risk of endocarditis. These findings hold important implications for the health of individuals with poor glycemic status.

## Conclusion

We characterized salivary microbiota and IgA responses associated with abnormal glycemic control in pre-diabetic individuals. As the salivary microbiota and IgA responses against it may influence glycemic control, further elucidation of their underlying mechanisms may help in the development of novel diagnostic and therapeutic approaches to reduce the risk of T2DM.

## Data Availability

The raw data supporting the conclusions of this article will be made available by the authors, without undue reservation.
