# Supplementary material for: Salivary microbiota and IgA responses are different in pre-diabetic individuals compared to normoglycemic controls
Source: Front Cell Infect Microbiol. 2025 Jun 4;15:1591285. doi: 10.3389/fcimb.2025.1591285 (PMC12174153; doi:10.3389/fcimb.2025.1591285)
Supplement: Supplementary file 1 [file Table1.docx]

Supplementary Material

**Supplementary Table 1.** Baseline characteristics and laboratory data of the study population (n = 38)

| **Variables** | **PreDM (n = 19)** | **Normal (n = 19)** | ***p*-value** |
| --- | --- | --- | --- |
| **Characteristics** |  |  |  |
| Age (years) | 75 (71–77) | 74 (71–77) | 0.948 |
| Sex (male, n (%)) | 4 (21%) | 6 (32%) | 0.714 |
| Hypertension ^a^ (n (%)) | 9 (47%) | 9 (47%) | > 0.999 |
| BMI (kg/m^2^) | 21.8 (19.8–23.0) | 21.2 (19.5–23.9) | 0.773 |
| Smoking status (n (%)) |  |  |  |
| Never smoker | 19 (100%) | 11 (58%) | 0.003 |
| Ex-smoker | 0 (0%) | 6 (32%) |  |
| Current smoker | 0 (0%) | 2 (11%) |  |
| **Oral condition** |  |  |  |
| Number of teeth (n (%)) |  |  |  |
| < 10 | 0 (0%) | 2 (11%) | 0.212 |
| 10-19 | 1 (5%) | 3 (16%) |  |
| ≥ 20 | 18 (95%) | 14 (74%) |  |
| Denture wearing (n (%)) | 4 (21%) | 7 (37%) | 0.476 |
| Salivary flow rate (g/min) | 1.7 (1.5–2.0) | 2.1 (1.8–2.9) | 0.028 |
| **Laboratory data** |  |  |  |
| HbA1c (NGSP, %) | 5.8 (5.7–5.9) | 5.3 (5.2–5.4) | < 0.001 |
| FPG (mg/dL) | 91 (90–99) | 90 (86–94) | 0.079 |

PreDM, prediabetes; BMI, body mass index; FPG, fasting plasma glucose.

Results are expressed as the median (1st–3rd quartiles) or percentage. *p*-values are obtained using Wilcoxon rank sum test or Fisher's exact test.

^a^ Hypertension is defined as blood pressure ≥ 140/90 mmHg or use of anti-hypertensive drugs.

**Supplementary Table 2.** The 20 most abundant genera in PreDM and Normal group

| **PreDM group (n=101)** | | **Normal group (n=101)** | |
| --- | --- | --- | --- |
| Genus | Mean relative abundance (%) | Genus | Mean relative abundance (%) |
| *Prevotella* | 22.12 | *Prevotella* | 20.90 |
| *Streptococcus* | 14.99 | *Streptococcus* | 16.63 |
| *Neisseria* | 7.55 | *Veillonella* | 6.63 |
| *Veillonella* | 6.51 | *Neisseria* | 6.32 |
| *Actinomyces* | 6.39 | *Porphyromonas* | 5.94 |
| *Porphyromonas* | 5.79 | *Actinomyces* | 5.77 |
| *Haemophilus* | 3.88 | *Fusobacterium* | 3.83 |
| *Fusobacterium* | 3.43 | *Rothia* | 3.64 |
| *Rothia* | 3.18 | *Haemophilus* | 3.05 |
| *Granulicatella* | 1.88 | Unidentified genus in *c_TM7-3* | 2.20 |
| Unidentified genus in *c_TM7-3* | 1.78 | *Granulicatella* | 1.66 |
| *Leptotrichia* | 1.48 | *Leptotrichia* | 1.51 |
| *Peptostreptococcus* | 1.46 | *Capnocytophaga* | 1.47 |
| *Capnocytophaga* | 1.43 | *Peptostreptococcus* | 1.38 |
| *Selenomonas* | 1.27 | *Selenomonas* | 1.31 |
| *Campylobacter* | 1.27 | *Campylobacter* | 1.17 |
| Unidentified genus in *c_TM7-3_o_CW040_f_F16* | 1.08 | Unidentified genus in *f_Streptococcaceae* | 0.98 |
| [Prevotella] | 1.04 | *Megasphaera* | 0.88 |
| *Parvimonas* | 0.90 | Unidentified genus in *c_TM7-3_o_CW040_f_F16* | 0.87 |
| *Megasphaera* | 0.88 | *Parvimonas* | 0.85 |

**
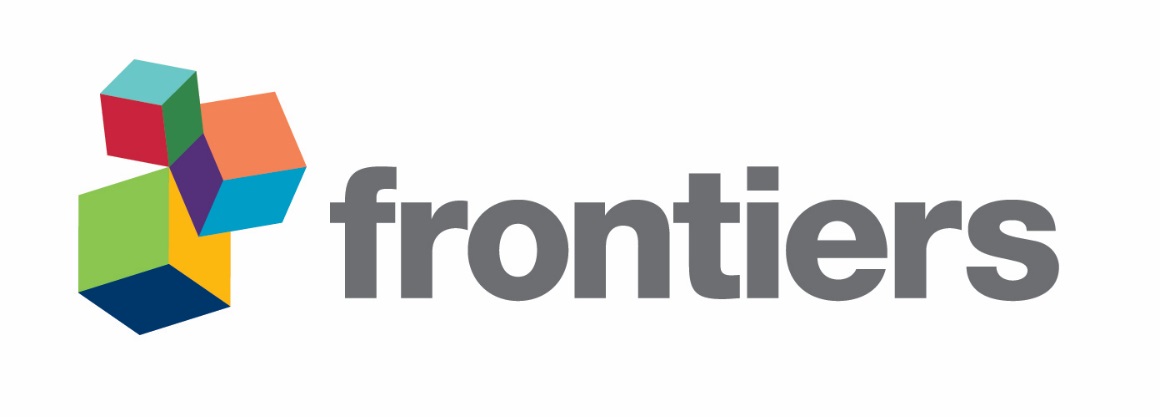
**
