# Supplementary figures and images for: Salivary microbiota and IgA responses are different in pre-diabetic individuals compared to normoglycemic controls
Source: Front Cell Infect Microbiol. 2025 Jun 4;15:1591285. doi: 10.3389/fcimb.2025.1591285 (PMC12174153; doi:10.3389/fcimb.2025.1591285)

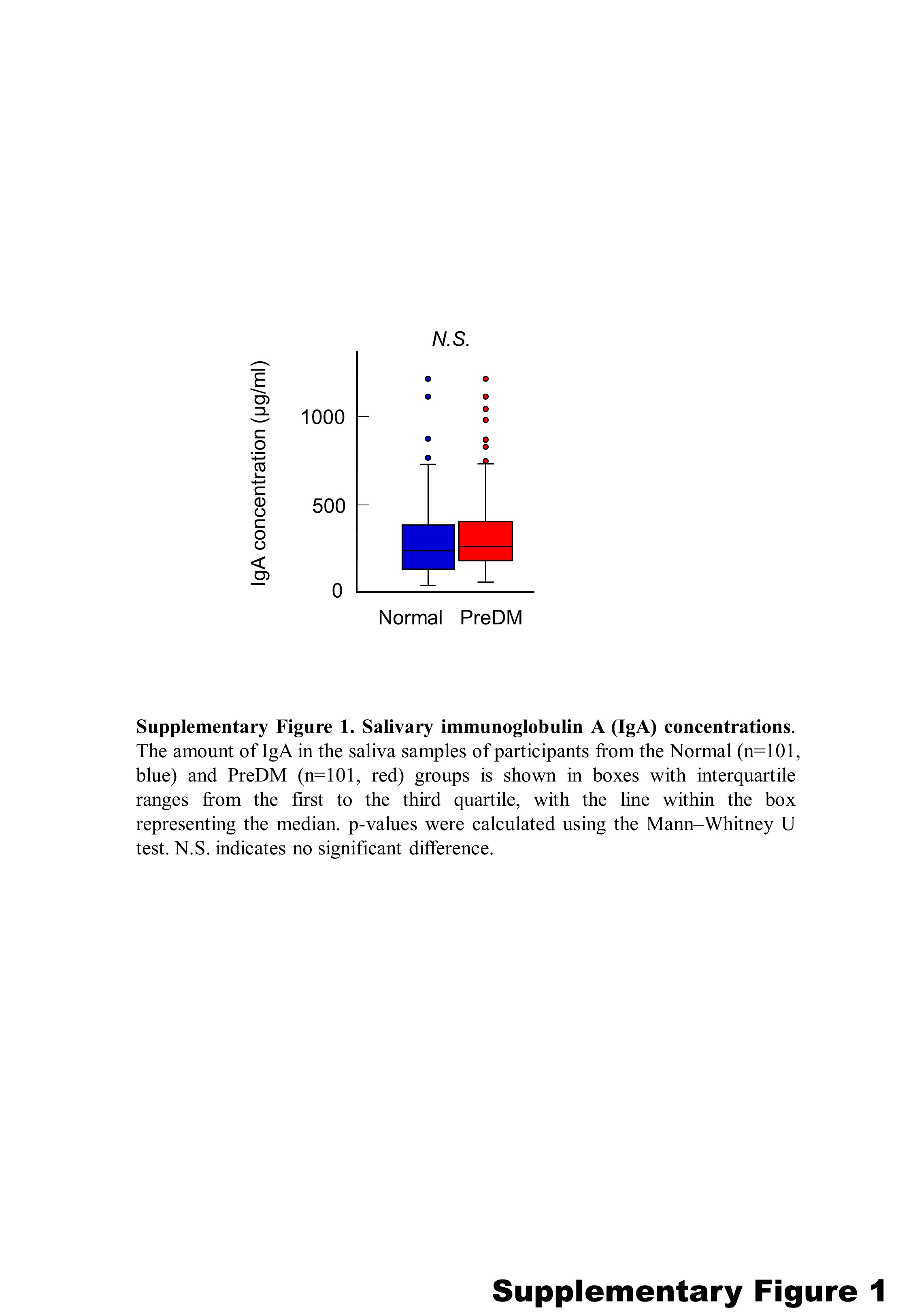

Supplement: Supplementary file 2 [file Image1.jpeg]
